# Supplementary material for: Pediatric Early Warning System (PEWS) Association with ICU Mortality in Children with Acute Lymphoblastic Leukemia: A Cohort Study from Kazakhstan
Source: Medicina (Kaunas). 2025 Nov 18;61(11):2054. doi: 10.3390/medicina61112054 (PMC12654657; doi:10.3390/medicina61112054)
Supplement: Supplementary file 1 [file medicina-61-02054-s001.zip › Supplementary Tables (S1-S7).pdf]

S1 Table. Detailed characteristics of the study participants

		PEWS use (No) Mean±SD / Frequency (%)	PEWS use (Yes) Mean±SD/ Frequency (%)	p-value
Clinical history and disease characteristics				
Blood type				0.04
	0(I)	58 (30.85)	45 (42.06)	
	A(II)	72 (38.30)	28 (26.17)	
	AB(IV)	18 (9.57)	5 (4.67)	
	B(III)	40 (21.28)	29 (27.10)	
Rh factor				0.14
	Positive	185 (98.40)	102 (95.33)	
	Negative	3 (1.60)	5 (4.67)	
ALL type				0.60
	B-lineage	138 (73.40)	77 (72.00)	
	T-lineage	44 (23.41)	24 (22.4)	
	Other	6 (3.19)	6 (5.61	
FAB				0.69
	L1	60 (32.09)	34 (31.78)	
	L2	122 (65.24)	72 (67.29)	
	L3	5 (2.67)	1 (0.93)	
Risk group				0.01
	High	104 (55.32)	43 (40.19)	
	Intermediate	32 (17.02)	17 (15.89)	
	Standard	52 (27.66)	47 (43.93)	
CNS leukemia				0.01
	No	135 (71.81)	92 (85.98)	
	Yes	53 (28.19)	15 (14.02)	
Relapse				0.04
	No	126 (67.02)	84 (78.5)	
	Once	43 (22.87)	18 (16.82)	
	Twice	19 (10.11)	4 (3.74)	
	Thrice	-	1 (0.93)	
Chemotherapy protocol				0.01
	AIEOP 2009	102 (54.26)	79 (73.83)	
	ALL REZ AIEOP 200900AIEOP 2009	59 (31.38)	23 (21.5)	
	INTERFANT	1 (0.53)	-	
	MLL-Baby AIEOP 2009006	23 (12.23)	5 (4.67)	
	NHL-BFM-2004	3 (1.6)	-	
Chemo days		15.02±16.37	13.08±13.39	0.30
Transfusion history				
Erythrocyte transfusion		4.21±2.81	4.6±3.63	0.36
Platelet transfusion		16.48±17.91	16.66±24.58	0.95
Plasma transfusion		6.13±6.45	7.35±11.43	0.34
Albumin transfusion		1.98±2.49	1.55±2.50	0.17
Vital signs when admitted to ICU				
Temperature (°C)		37.21±0.81	37.09±0.61	0.15
Number of hyperthermia episodes within 24 hours of ICU admission	0	87 (52.10)	57 (53.27)	0.10

	1	20 (11.98)	11 (10.28)	
	2	37 (22.16)	31 (28.97)	
	3	14 (8.38)	8 (7.48)	
	4	9 (5.39)	-	
Heartrate (beats/min)		112.74±18.59	110.61±16.84	0.33
Systolic blood pressure (mmHg)		95.36±10.45	94.81±10.89	0.68
Diastolic blood pressure (mmHg)		57.63±8.87	58.08±8.71	0.68
Breath rate (breath/min)		25.18±6.52	25.28±6.18	0.90
Saturation ()		96.27±3.57	96.93±3.09	0.11
Clinical symptoms when admitted to ICU				
SIRS				<0.001
	No	155 (82.45)	90 (84.11)	
	Yes	33 (17.55)	17 (15.89)	
Cough				0.69
	No	144 (85.71)	89 (83.18)	
	Yes	24 (14.29)	18 (16.82)	
Hemorrhagic syndrome	No			0.01
	Yes	112 (59.57)	81 (75.70)	
		76 (40.43)	26 (24.30)	
Cytopenia				0.26
	No	100 (53.19)	65 (60.75)	
	Yes	88 (46.81)	42 (39.25)	
Laboratory test results when admitted to ICU				
Hemoglobin (g/L)		86.42±16.25	89.69±18.88	0.14
Erythrocyte (10^3 cells/μL)		2.99±0.56	3.02±0.50	0.67
White cells (10^3 cells/μL)		24.85±86.23	55.66±155.00	0.06
Absolute neutrophil count (10^3 cells/μL)		1.5±3.16	2.34±3.39	0.04
Thrombocytes (10^3 cells/μL)		54.12±74.41	68.67±86.41	0.15
ESR (mm/hour)		23.53±10.89	23.74±13.54)	0.89
ALT (U/L)		98.87±153.74	42.62±73.68	<0.0001
AST (U/L)		78.62±152.05	42.83±43.84	0.00
Bilirubin (μmol/L)		23.97±30.37	18.49±20.1	0.07
Direct bilirubin (μmol/L)		14.95±22.5	10.51±16.27	0.06
Glucose (mmol/L)		5.71±3.32	4.74±0.96	<0.0001
Creatinine (μmol/L)		64.16±103.28	54.27±34.50	0.25
Urea (mmol/L)		7.07±5.61	6.07±3.81	0.08
Total protein (g/L)		53.83±8.51	58.88±7.74	0.85
CRP (mg/L)		79.39±89.97	82.49±96.72	<0.001
Sodium (mmol/L)		136.27±3.75	136.83±3.53	0.79
Potassium (mmol/L)		3.98±0.81	3.92±0.67	0.21
Calcium (mmol/L)		2.06±0.23	2.18±0.25	0.52
Chlorides (mmol/L)		102.5±5.46	103.46 - ±5.37	<0.001
APTT (sec)		42.39±10.7	31.68±8.85	0.15
PTI ()		73.42±17.89	80.21±22.64	<0.00
PT (sec)		14.59±2.66	13.19±2.53	0.01
INR		1.22±0.24	1.18±0.25	<0.0001
Fibrinogen (g/L)		2.8±1.3	2.93±1.20	0.14
Urine specific gravity		1013.27±4.2	1011.64±4.89	0.40

Urine pH	5.93±0.9	6.17±0.90	<0.001
Proteinuria (g/L)	0.04±0.15	0.08±0.21	0.03
Instrumental examination results			
Splenomegaly			
No	48 (28.57)	14 (13.08)	
Yes	120 (71.43)	93 (86.92)	<0.0001
Hepatomegaly			
No	4 (2.38)	-	
Yes	164 (97.62)	107 (100)	0.16
Lymphadenopathy			
No	47 (27.98)	23 (21.50)	
Yes	121 (72.02)	84 (78.50)	0.29
Pancreatic enlargement			
No	131 (77.98)	61 (57.55)	
Yes	37 (22.02)	45 (42.45)	<0.001
Pleural effusion			
No	153 (91.07)	102 (95.33)	
Yes	15 (8.93)	5 (4.67)	0.28
Ascitic fluid			
No	157 (93.45)	105 (99.06)	
Yes	11 (6.55)	1 (0.94)	0.03
ECG			
Tachycardia	94 (55.95)	46 (42.99)	
Arrhytmia	3 (1.79)	-	
Bradycardia	3 (1.79)	1 (0.93)	
Norm	68 (40.48)	60 (56.07)	0.04
EchoCG			
Norm	129 (76.79)	93 (86.92)	
Pericarditits	15 (8.93)	3 (2.80)	
Reduced ejection fraction	22 (13.10)	8 (7.48)	
Ventricular hypertrophy	2 (1.19)	3 (2.80)	0.05
Chest X-ray			
Norm	99 (58.93)	78 (72.90)	
Bilateral pneumonia	39 (23.21)	15 (14.02)	
Left side pneumonia	16 (9.52)	5 (4.67)	
Lung abscess	1 (0.6)	-	
Pneumothorax with pneumonia	1 (0.6)	1 (0.93)	
Right side pneumonia	12 (7.14)	8 (7.48)	0.09

Abbreviations: AAL – acute lymphoblastic leucosis; ALT - Alanine aminotransferase; APTT - Activated partial thromboplastin time; AST - Aspartate aminotransferase; BMI – body mass index; CNS – central nervous system; CRP - C-reactive protein; ECG – electrocardiogram; EchoCG – echocardiogram; ESR – Erythrocyte sedimentation rate; ICU – intensive care unit; INR - International normalized ratio; LoS – length of stay; PI - Prothrombin index; PT - Prothrombin time; PEWS – pediatric early warning score; Rh factor – Rhesus factor; SIRS - systemic inflammatory response syndrome.

S2 Table. Univariable and Multivariable Logistic Regression Analyses for Demographic and General Characteristics

Characteristics		OR (95% CI)	p-value	AOR (95% CI)	p-value
Age		1.02 (0.97 - 1.08)	0.40		
Gender (Ref. Female)					
	Male	0.42 (0.24 - 0.74)	0.002	0.50 (0.25 - 0.99)	0.04
Ethnicity (Ref. Kazakh)					
	Russian	2.37 (0.83 - 6.35)	0.09		
	Other	0.85 (0.35 - 1.88)	0.70		
BMI (Ref. Normal weight)					
	Underweight	1.70 (0.92 - 3.13)	0.09		
	Overweight	1.76 (0.67 - 4.29)	0.22		
ICU readmission (same hospitalization) (Ref. 1)					
	2	1.65 (0.74 - 3.51)	0.20		
	3	0.60 (0.03 - 3.61)	0.63		
ICU readmission (different hospitalizations) (Ref. 0)					
	1	1.92 (0.82 - 4.25)	0.12		
	2	2.81 (0.36 - 17.47)	0.26		
	3	3.87 (0.45 – 67.86)	0.98		
Days in hospital		0.95 (0.93 - 0.96)	<0.001	0.92 (0.89 - 0.95)	<0.001
LoS before ICU		1.01 (0.99 - 1.02)	0.26		
LoS in ICU		1.05 (1.02 - 1.10)	<0.01	1.15 (1.09 - 1.22)	<0.001
PEWS use (Ref. No)					
	Yes	0.52 (0.28 - 0.94)	0.04	0.89 (0.38 - 1.98)	0.76

The reference category for the dependent variable, *mortality*, is "No."

Abbreviations: AOR – adjusted odds ratio; BMI – body mass index; ICU – intensive care unit; LoS – length of stay; OR – odds ratio; PEWS – pediatric early warning score.

S3 Table. Univariable and Multivariable Logistic Regression Analyses for Clinical History and Disease Characteristics

Characteristics		OR (95% CI)	p-value	AOR (95% CI)	p-value
Blood type (Ref. 0(I))					
	A(II)	1.04 (0.55 - 1.97)	0.90		
	AB(IV)	1.10 (0.36 - 2.98)	0.86		
	B(III)	0.59 (0.26 - 1.27)	0.19		
Rh factor (Ref. Positive)					
	Negative	0.88 (0.20 - 6.09)	0.88		
ALL type (Ref. B-lineage)					
	T-lineage	1.67 (0.08 - 18.05)	0.68		
	Other	1.18 (0.52 - 2.55)	0.69		
FAB (Ref. L1)					
	L2	0.87 (0.47 - 1.56)	0.64		
	L3	0.01 (0.05 - 119.00)	0.99		
Risk group (Ref. High)					
	Intermediate	0.49 (0.21 - 1.06)	0.09	0.68 (0.28 - 1.55)	0.38

Standard	0.30 (0.15 - 0.59)	<0.001	0.42 (0.19 - 0.88)	0.02
CNS leukemia (Ref. No)				
Yes	2.33 (1.27 - 4.23)	0.01	0.93 (0.43 - 1.98)	0.86
Relapse (Ref. No)				
Once	4.55 (2.44 - 8.55)	<0.001	3.61 (0.70 - 20.86)	0.12
Twice	1.89 (0.64 - 4.94)	0.21	0.00 (0 - 1.22)	1.00
Thrice	0.00 (0.01 - 218.12)	0.99	1.29 (0.19 - 9.55)	0.79
Chemotherapy protocols (Ref. AIEOP 2009)	3.50 (1.93 - 6.41)	<0.001	1.12 (0.20 - 5.66)	0.89
ALL REZ AIEOP 200900AIEOP 2009	314.3 (0.01 - 568.90)	0.99	51.89 - (0.01 – 856.1)	0.99
INTERFANT	1.49 (0.51 - 3.82)	0.43	1.19 (0.39 - 3.17)	0.74
MLL-Baby AIEOP 2009006	0.00 (0.01 - 3.60)	0.99	0.00 (- 4.72)	0.99
NHL-BFM-2004				
Chemo days	1.01 (0.99 - 1.03)	0.22		

The reference category for the dependent variable, *mortality*, is "No."

Abbreviations: AAL – acute lymphoblastic leucosis; AOR – adjusted odds ratio; CNS – central nervous system; FAB - French-American-British classification; OR – odds ratio.

S4 Table. Univariable and Multivariable Logistic Regression Analyses for Vital Signs When Admitted to ICU

Characteristics	OR (95% CI)	p-value	AOR (95% CI)	p-value
Temperature	1.23 (0.85 - 1.76)	0.28		
Number of hyperthermia episodes				
within 24 hours of ICU admission (Ref.	1.22 (0.47 - 2.89)	0.67	1.16 (0.44 - 2.84)	0.76
0)	0.99 (0.48 - 1.96)	0.98	0.85 (0.40 - 1.73)	0.65
1	1.03 (0.32 - 2.84)	0.96	0.82 (0.24 - 2.39)	0.73
2	7.00 (1.75 - 34.65)	0.01	5.42 (1.28 - 27.79)	0.03
3				
4				
Heartrate	1.03 (1.01 - 1.04)	<0.001	1.02 (1.00 - 1.04)	0.02
Systolic blood pressure	1.01 (0.98 - 1.04)	0.46		
Diastolic blood pressure	1.01 (0.98 - 1.04)	0.66		
Breath rate	1.04 (0.99 - 1.08)	0.09		
Saturation	0.90 (0.83 - 0.97)	0.01	0.93 (0.85 - 1.01)	0.06

The reference category for the dependent variable, *mortality*, is "No."

Abbreviations: AOR – adjusted odds ratio; OR – odds ratio.

S5 Table. Univariable and Multivariable Logistic Regression Analyses for Clinical Symptoms When Admitted to ICU

Characteristics	OR (95% CI)	p-value	AOR (95% CI)	p-value
SIRS (Ref. No)				
Yes	6.72 (3.51 – 13.10)	<0.001	4.61 (2.25 – 9.61)	<0.001
Cough (Ref. No)				
Yes	2.25 (1.11 - 4.49)	0.02	2.36 (1.12 - 4.90)	0.02
Hemorrhagic syndrome (Ref. No)				
Yes	1.90 (1.09 - 3.32)	0.02	1.55 (0.84 - 2.83)	0.16
Cytopenia (Ref. No)				

Yes	3.16 (1.80 - 5.67)	<0.001	2.52 (1.39 - 4.64)	<0.001

The reference category for the dependent variable, *mortality*, is "No."

Abbreviations: AOR – adjusted odds ratio; OR – odds ratio; SIRS - systemic inflammatory response syndrome.

S6 Table. Univariable and Multivariable Logistic Regression Analyses for Laboratory Test Results When Admitted to ICU

Characteristics	OR (95% CI)	p-value	AOR (95% CI)	p-value
Hemoglobin	0.99 (0.98 - 1.01)	0.51		
Erythrocyte	1.01 (0.60 - 1.68)	0.97		
White cells	0.99 (0.98 - 1.00)	0.08		
Absolute neutrophil count	0.82 (0.69 - 0.94)	0.01	0.92 (0.78 - 1.05)	0.31
Thrombocytes	1.00 (0.99 - 1.00)	0.17		
ESR	1.02 (0.99 - 1.04)	0.16		
ALT	1.00 (1.00 - 1.00)	0.20		
AST	1.00 (1.00 - 1.00)	0.25		
Bilirubin	1.00 (0.99 - 1.01)	0.39		
Direct bilirubin	1.01 (0.99 - 1.02)	0.30		
Glucose	1.10 (1.00 - 1.21)	0.049	1.09 (0.98 - 1.22)	0.14
Creatinine	0.98 (0.97 - 1.00)	0.03	0.98 (0.96 - 1.00)	0.06
Urea	1.02 (0.97 - 1.08)	0.35		
Total protein	0.95 (0.92 - 0.98)	<0.001	1.00 (0.96 - 1.04)	0.93
CRP	1.01 (1.00 - 1.01)	<0.001	1.00 (1.00 - 1.01)	0.04
Sodium	0.95 (0.88 - 1.02)	0.18		
Potassium	0.88 (0.60 - 1.26)	0.49		
Calcium	0.69 (0.22 - 2.09)	0.51		
Chlorides	1.02 (0.97 - 1.07)	0.44		
APTT	1.06 (1.03 - 1.09)	<0.001	1.04 (1.00 - 1.07)	0.04
PTI	0.98 (0.97 - 1.00)	0.01	1.00 (0.96 - 1.03)	0.91
PT	1.14 (1.04 - 1.27)	0.01	0.87 (0.59 - 1.26)	0.46
INR	3.93 (1.35 - 11.70)	0.01	7.18 (0.08 - 737.71)	0.40
Fibrinogen	0.91 (0.72 - 1.14)	0.42		
Urine specific gravity	1.00 (0.94 - 1.06)	0.90		
Urine pH	0.99 (0.72 - 1.33)	0.93		
Proteinuria	2.27 (0.51 - 9.10)	0.25		

The reference category for the dependent variable, *mortality*, is "No."

Abbreviations: ALT - Alanine aminotransferase; AOR – adjusted odds ratio; APTT - Activated partial thromboplastin time; AST - Aspartate aminotransferase; CRP - C-reactive protein; ESR – Erythrocyte sedimentation rate; INR - International normalized ratio; OR – odds ratio; PI - Prothrombin index; PT - Prothrombin time; Rh factor – Rhesus factor.

S7 Table. Univariable and Multivariable Logistic Regression Analyses for Instrumental Examination Results When Admitted to ICU

Characteristics		OR (95% CI)	p-value	AOR (95% CI)	p-value
Splenomegaly (Ref. No)					
	Yes	1.25 (0.64 - 2.56)	0.53		

Hepatomegaly (Ref. No)				
Yes	185.86 (0.00 – 8887)	0.98		
Lymphadenopathy (Ref. No)				
Yes	1.36 (0.71 - 2.72)	0.37		
Pancreatic enlargement (Ref. No)				
Yes	1.02 (0.55 - 1.85)	0.94		
Pleural effusion (Ref. No)				
Yes	2.26 (0.85 - 5.74)	0.09		
Ascitic fluid (Ref. No)				
Yes	2.35 (0.68 - 7.64)	0.16		
ECG (Ref. Tachycardia)				
Arrhythmia	4.36 (0.41 - 95.37)	0.23	5.10 (0.41 - 120.28)	0.21
Bradycardia	0.73 (0.04 - 5.86)	0.79	1.31 (0.06 - 12.05)	0.83
Norm	0.38 (0.20 - 0.69)	<0.001	0.55 (0.27 - 1.12)	0.10
EchoCG (Ref. Norm)				
Pericarditits	3.75 (1.35 - 10.13)	0.01	2.30 (0.72 - 7.10)	0.15
Reduced ejection fraction	6.14 (2.77 - 13.91)	<0.001	3.03 (1.20 - 7.71)	0.02
Ventricular hypertrophy	3.13 (0.40 - 19.48)	0.22	2.54 (0.28 - 18.92)	0.36
Chest X-ray (Ref. Norm)				
Bilateral Pneumonia	10.02 (5.06 - 20.45)	<0.001	7.45 (3.61 - 15.74)	<0.001
Left side pneumonia	1.50 (0.41 - 4.48)	0.50	0.81 (0.19 - 2.69)	0.75
Lung abscess	0.00	0.99	0.00	0.99
Pneumothorax	0.00	0.99	0.00	0.99
Right side pneumonia	2.13 (0.64 - 6.07)	0.18	1.34 (0.35 - 4.29)	0.64

The reference category for the dependent variable, *mortality*, is "No."

Abbreviations: AOR – adjusted odds ratio; ECG – electrocardiogram; EchoCG – echocardiogram; OR – odds ratio.
